# Non-autistic observers both detect and demonstrate the double empathy problem when evaluating interactions between autistic and non-autistic adults

**DOI:** 10.1177/13623613231219743

**Published:** 2023-12-27

**Authors:** Desiree R Jones, Monique Botha, Robert A Ackerman, Kathryn King, Noah J Sasson

**Affiliations:** 1 The University of Texas at Dallas, USA; 2 University of Stirling, UK

**Keywords:** double empathy, social cognition and social behavior, social interaction

## Abstract

Consistent with a “double empathy” framework, autistic adults often experience better interaction with autistic compared with non-autistic partners. Here, we examined whether non-autistic observers detect differences in autistic interactions relative to non-autistic and mixed ones. Non-autistic adults (*N* = 102) rated the interaction quality and traits of 42 autistic and 44 non-autistic male participants interacting in same or mixed-neurotype dyads. Non-autistic interactions and participants were evaluated most positively, with participants rated more favorably when interacting with non-autistic partners and rated as less intelligent and awkward when interacting with autistic partners. Observers perceived mixed interactions as the least successful overall. Whereas non-autistic interactions were rated as smoother and more enjoyable than mixed interactions, they were not rated differently from autistic interactions on any measure of interaction quality. Observers also perceived that non-autistic participants but not autistic participants disclosed more to non-autistic partners. However, they evaluated autistic participants more negatively than their partners in the interaction evaluated them; they disproportionately underestimated trust and intelligence ratings made by autistic participants; and they and reported lower social interest in participants than did the autistic and non-autistic people in the interactions. Collectively, these findings indicate that non-autistic adults both detect and demonstrate the double empathy problem when observing social interactions involving autistic people.

**Lay Abstract:**

The “double empathy problem” refers to breakdowns in communication and understanding that frequently occur between autistic and non-autistic people. Previous studies have shown that autistic people often establish better rapport and connection when interacting with other autistic people compared to when interacting with non-autistic people, but it is unclear whether this is noticeable to non-autistic observers. In this study, 102 non-autistic undergraduate students viewed and rated video recordings of “get to know you” conversations between pairs of autistic and non-autistic adults. Sometimes the pairs were two autistic people, sometimes they were two non-autistic people, and sometimes they were “mixed” interactions of one autistic and one non-autistic person. Observers tended to rate non-autistic participants and their interactions the most favorably, but—consistent with the “double empathy problem”—they rated mixed interactions between autistic and non-autistic people as the least successful. They also perceived that only non-autistic people disclosed more when interacting with a non-autistic conversation partner. Autistic participants’ partners in the conversations tended to evaluate them more favorably than did outside observers, suggesting that personal contact may facilitate more positive evaluations of autistic people. Furthermore, observers expressed less social interest in participants than did the autistic and non-autistic participants in the interactions. Together, these findings suggest that non-autistic observers both detect and demonstrate some aspects of the double empathy problem.

## Introduction

Autistic people are a socially marginalized minority group who are exposed to processes of social stigma and minority stress (Botha & Frost, 2020). Their social presentations, behavior, and communication tend to fall outside of societal norms and are often stigmatized by non-autistic (NA) people (Butler & Gillis, 2011; Cage et al., 2019; Gillespie-Lynch et al., 2021; Johnson & Joshi, 2016; Jones, DeBrabander, et al., 2021). Not coincidentally, autistic adults often experience worse social outcomes than NA adults (Mitchell et al., 2021), including self-reporting high levels of unwanted loneliness in both quantitative and qualitative studies (Ee et al., 2019; Mazurek, 2014; Umagami et al., 2022) and lower quality of life^1^ across the lifespan (Barneveld et al., 2013; Eaves & Ho, 2008; Howlin et al., 2004; Levy & Perry, 2011; Seltzer et al., 2004). Importantly, autistic traits do not predict quality of life (van Heijst & Geurts, 2015), which suggests that need-environment fit and right to self-determination (Kapp, 2018) rather than autistic characteristics are primary contributors.

### Deficit frameworks of autism

Autism research and practice has traditionally attributed these poor social outcomes to autistic “deficits” (Kapp et al., 2013). Within this medical model approach, treatment for social disability commonly focuses on trying to train autistic people to be more normative in their social thinking and behavior, a process that can teach autistic people to “mask” their innate social behavior, despite immense effort or impact of masking (Pearson & Rose, 2021). These programs are also limited in their effect (Bishop-Fitzpatrick et al., 2014; Gates et al., 2017; Palmen et al., 2010; Williams White et al., 2007): those found for social skills and social cognitive training programs are often restricted to the study environment (e.g. improved social knowledge or task performance) and do not commonly translate to real-world social outcomes for autistic adults (Gates et al., 2017; Rao et al., 2008). This may occur in part because this approach fails to account for real world context or acknowledge that outcomes for marginalized minorities are shaped by wider experiences within society. Furthermore, it often ignores the stigma and additional stress burden autistic people face trying to navigate non-autistic spaces. Finally, what behavior is considered socially acceptable and normative is often a moving target that shifts based on context, culture, and relational dynamics (Milton, 2017), making it difficult for autistic people to navigate and harmful to mental well-being to be forced to adopt non-autistic conventions (Pearson & Rose, 2021).

### The double empathy problem

This stigma and stress can be reduced in autistic company. Autistic people often have better social interaction experiences with other autistic people than they do with NA partners. They communicate, establish rapport, and develop social interest with each other just as effectively as NA people do (e.g. Chen et al., 2021; Crompton, Sharp, et al., 2020; Crompton, Hallett, et al., 2020; Granieri et al., 2020; Heasman & Gillespie, 2019; Morrison et al., 2020). It is specifically within interactions between autistic and NA people that communication and rapport deteriorates (Crompton, Ropar, et al., 2020; Morrison et al., 2020). Such findings challenge social deficit models of autism and instead support a “double empathy” framework for understanding social disability in autism (Milton, 2012). In contrast to a deficit model that attributes poor cross-neurotype interactions exclusively to the autistic person (and the onus on them to change to improve them), a double empathy approach emphasizes that social interactions are bi-directional with both parties equally contributing to poor outcomes. Autistic and NA people differ in their communication styles, social preferences, and expectations, and these differences contribute to relational breakdowns in social connection and understanding (Crompton et al., 2021).

### The effects of the double empathy problem for autistic people

In recent years, an emerging literature has taken a more dynamic approach to understanding social outcomes for autistic people by examining how stigma, lack of acceptance, minority stress, negative social judgments, and dehumanization by NA adults affect outcomes for autistic people (Botha & Frost, 2020; Cage et al., 2018, 2019; Morrison et al., 2020; Sasson et al., 2017; Stevanovic et al., 2017). Part of this growing literature has investigated how stigma and social judgments affect the interpersonal and relational outcomes between autistic and NA people (Alkhaldi et al., 2021; DeBrabander et al., 2019; Morrison et al., 2019, 2020; Sasson et al., 2017; Sasson & Morrison, 2019). For example, NA people often misunderstand autistic peoples’ mental states, intentions, and behaviors (Alkhaldi et al., 2019; Edey et al., 2016; Sheppard et al., 2016), just as autistic people often misread NA social cues (for a review, see Sasson et al., 2011). Compounding this problem, autistic adults also face barriers to social inclusion in NA environments before social interaction even begins. NA adults rapidly form negative first impressions of autistic adults that are strongly associated with reduced social interest in autistic partners (Cage et al., 2019; DeBrabander et al., 2019; Jones, Morrison, et al. 2021; Morrison et al., 2019, 2020; Sasson et al., 2017; Scheerer et al., 2022; Stagg et al., 2014). These impressions are driven by unfavorable evaluations of autistic social presentations and behavior, as they do not occur when only reading transcripts of autistic communication (Sasson et al., 2017). Collectively, these studies support a reconceptualization of autistic sociality around a double empathy framework emphasizing the relational breakdown in social understanding between autistic and non-autistic people rather than an inherent deficit within autistic people (Milton, 2012).

Interrelational dynamics are not only limited to interactions between two people but also include evaluations of these interactions from outside observers (Wall et al., 2018; Wilson et al., 2022). Observers of interactions often form judgments not only about the interaction itself (such as smoothness and participant rapport) but also about each person within an interaction. It remains unclear, however, whether interactions between autistic and non-autistic people are evaluated differently by NA observers. Do they detect reduced social quality between autistic and NA partners relative to NA-NA and autistic-autistic interactions, as has been found in prior studies (Crompton, Ropar, et al., 2020; Crompton, Sharp, et al., 2020; Morrison et al., 2020)? If so, does this correspond with poorer impressions of the partners within the interaction, particularly the autistic partner who may socialize in non-normative ways and be perceived by NA people as the cause of the disjunction in the interaction? Undervaluing autistic sociality relative to non-autistic social norms and disproportionately attributing social disjuncture in mixed interactions to the autistic participant can contribute to social stigmatization of autistic people and increase potential for real-world exclusion, victimization, and discrimination (Crompton et al., 2021; Mitchell et al., 2021). For example, autistic people have been misperceived by NA observers in criminal justice settings as deceptive and lacking credibility (Lim et al., 2022), in job interview settings as less competent and hirable (Whelpley & May, 2023), in the workplace as misbehaving (Szechy et al., 2023), and in peer settings as not as socially motivated as they actually are (Black et al., 2022).

## The current study

In this study, NA observers evaluated the social interaction quality of previously recorded videos of conversations between autistic partners, NA partners, and “mixed” interactions of autistic and NA partners (see Morrison et al., 2020). In the prior study (Morrison et al., 2020), NA participants in the interactions expressed greater social interest in NA relative to autistic partners, but autistic participants did not share this preference and instead trended toward greater interest in other autistic partners. Autistic participants also reported disclosing more about themselves when interacting with another autistic person.

Here, we predicted that NA social behavior and communication styles would be privileged by NA observers, which would manifest in more favorable ratings of NA-NA interactions relative to Autistic-Autistic (A-A) and A-NA ones, and more favorable ratings of NA participants relative to autistic ones. We also explored whether NA observers detected enhanced social quality in A-A relative to A-NA interactions, as the participants in Morrison et al. (2020) previously reported experiencing. If not, this might indicate the imposition of normative standards to evaluations of autistic interactions and reflect a double empathy failure in recognizing signifiers of autistic sociality. Finally, we compared ratings of NA observers to the ratings provided by the participants within the interaction. Consistent with the double empathy framework, we predicted that NA observers would privilege NA ways of socializing and their ratings would better align with those provided by NA compared to autistic participants in the interaction. Specifically, we predicted that NA observers would misjudge autistic participants’ perceptions of their partner and the quality of the interaction to a greater degree than NA participants’ perceptions.

## Method

### Participants

Autistic participants were recruited from the Autism Research Collaborative at UT Dallas, a registry of local autistic adults who have consented to be contacted about research opportunities. These autistic adults have all scored above the clinical threshold for ASD on the Autism Diagnostic Observation Schedule (ADOS-II; Lord et al., 2012). Only those with WASI full-scale intelligence quotients of 90 or above were recruited for this study to aid in matching with NA participants, who were recruited from the university subject pool and from a database of previous participants who had consented to be contacted for future research opportunities. Autistic and NA participants did not significantly differ on self-reported gender identity (all male), race (Autistic: 81.4% White; NA: 79.1% White, *p* = 0.39), education (Autistic: 80% at least some college, NA: 88% at least some college, *p* = 0.25), maternal education (Autistic: 89% at least some college, NA = 76% at least some college, *p* = 0.06) or IQ as estimated by the WRAT-3 (Wilkinson, 1993) reading subtest (Autistic: *M* = 109.86, *SD* = 8.60; NA: *M* = 111.51, *SD* = 7.70; *p* = 0.35) but did differ on age (Autistic: *M* = 23.84, *SD* = 3.93; NA: *M* = 20.77, *SD* = 3.27; *p* < 0.001).

Videos of these conversations were then shown to 102 NA observers (92 self-identified as female, M_AGE_: 20.37 years, estimated M_IQ_: 110.55) recruited from the university subject pool who participated for course credit. All NA observers self-reported that they were not on the autism spectrum. Participants from both phases provided informed consent prior to beginning the study, and the protocol for this project was approved by the university institutional review board.

### Community involvement statement

This work was conceived, developed, conducted, interpreted, and written by a team of autistic, neurodivergent, and non-autistic researchers.

### Procedure

Autistic and NA adults were video recorded while participating in a 5 minute unstructured “get to know you” conversation with an unfamiliar autistic or NA partner in one of three dyadic conditions: both NA partners (NA-NA; *n* = 14), both autistic partners (A-A; *n* = 16), or one autistic partner and one non-autistic partner (A-NA; *n* = 13). This interaction procedure was initially developed to assess interactions between NA participants but has since been used with autistic participants (Morrison et al., 2020; Usher et al., 2018).

Observer participants were randomly assigned to view and evaluate interaction videos in one of the three dyadic conditions. Videos were presented one at a time using Qualtrics software, and each was followed by evaluation measures (detailed below). Participants were prevented from completing the measures until the video had finished playing and could not move on to the next video until completing every item on each measure. Observers were blinded to the diagnostic status of the conversation participants.

### Measures

Observers completed the Social Interaction Evaluation Measure (SIEM; Berry & Hansen, 1996) and the First Impression Scale (FIS; Sasson et al., 2017). Both measures were previously administered to the conversation participants themselves (for results, see Morrison et al., 2019) and here were rephrased to assess observer perceptions of the interaction and their impressions of each conversation partner. Using the same measures across studies also enabled additional exploratory analyses comparing observer and participant ratings.

The SIEM is a self-report questionnaire consisting of 10 items rated from 1 (“Not at all”) to 8 (“Very much”) on a Likert-type scale. The SIEM assesses perceptions of interaction quality among partners, and here was adapted for observer perceptions. The four items assessing perceptions of overall interaction quality on enjoyment, smoothness, awkwardness, and intimacy were phrased identically to those given to participants in the interaction (e.g. “to what extent was the interaction intimate?”). Second, the SIEM items in which interaction participants rate their partner were expanded so that the observer rated their perception of both interaction participants. The observers rated how much they thought each interaction participant: enjoyed the interaction, was satisfied with the interaction, found the interaction pleasant, influenced the interaction, desired future interaction with their partner, and disclosed in the interaction (e.g. “how much did person 1 disclose to person 2?”).

The FIS consists of 10 items in which participants rate their first impressions of conversation participants using a 4 scale ranging from “strongly disagree” to “strongly agree.” Six of the items correspond to different character traits (awkwardness, attractiveness, trustworthiness, dominance, likeability, and intelligence) and four correspond to their own social interest toward the participant (I would live near, hang out with, sit next to, and have a conversation with this person). Here, observers provided separate FIS ratings for each conversation partner. Items on the FIS are phrased identically whether completed by interaction participants or observers (e.g. “this person is likable”). In previous studies, NA observers have reliably rated autistic participants less favorably than NA comparison participants on the FIS (DeBrabander et al., 2019; Flower et al., 2021; Jones, DeBrabander, et al., 2021; Morrison et al., 2019; Sasson et al., 2017; Sasson & Morrison, 2019; Scheerer et al., 2022).

### Analytic strategy

Analyses occurred in two parts. The first part used multilevel modeling (MLM) with Restricted Maximum Likelihood Estimation (REML) to investigate the effects of dyad composition (NA-NA vs A-NA vs A-A) on observers’ ratings of overall interaction quality and their averaged perceptions of interaction participants’ experiences. A Bonferroni correction was used when following up any significant effects. Because observers provided ratings on multiple dyads, we included a random intercept for observers. We then used MLM with REML to evaluate the effects of the diagnostic status of both interaction partners on observers’ perceptions of them and their experiences. We tested whether observers rated interaction participants differently depending upon the interaction participants’ own diagnosis and/or the diagnosis of their partner. Including an interaction term between both partners’ diagnostic statuses enabled us to test whether observer perceptions differed for autistic and NA partners in mixed versus same-neurotype interactions. Given that observers provided ratings on both interaction partners across multiple dyads, we included a random intercept for observers and permitted observers’ ratings across both interaction partners within dyads to correlate.

In the second part, we examined whether and how observer perceptions differed from those previously reported by the participants in the dyad (see Morrison et al., 2020). We used the Truth and Bias Model of Judgment framework (West & Kenny, 2011) to evaluate discrepancies and correspondences between these two sets of perceptions. The Truth and Bias model posits that judgments of phenomenon (e.g. observers’ ratings of the smoothness of the interaction) are pulled toward the truth (e.g. the interaction participants’ ratings of smoothness) and different forms of bias (e.g. a tendency for observers to rate interactions as less smooth). When the judgment and truth variables are both assessed on the same scales, these variables can be centered using the grand-mean of the truth variable prior to regressing the judgment variable on the truth variable. The intercept from this regression then reflects *directional bias* (i.e. the mean-level difference between the judgment and the truth), and the slope for the truth variable reflects *tracking accuracy* (i.e. the degree of rank-order consistency between the judgments and the truth). For this study, we treated observers’ ratings as the judgment variables and the interaction participants’ ratings as the truth variables. The grand-means for the truth variables were used to center the corresponding judgment and truth variables in each analysis.

Importantly, inferences about bias and accuracy are only warranted when observers and interaction participants provided ratings on the same person or dyad, as was the case for most items in our study (e.g. both rate the awkwardness of the interaction partners). However, some items (e.g. “this person is probably as smart as I am” and those assessing social interest on the FIS) have different reference points between observers and interaction participants, which can affect interpretation. For example, observers and interaction participants rated their own social interest toward the partner rather than rating the interaction participants’ social interest toward the partner. Therefore, we describe results for these items as mean-level differences and rank-order consistency rather than as directional bias and tracking accuracy, which allows us to evaluate whether the observers’ social interest aligns with interaction participants’ own social interest.

We used single-level multiple regression analyses to evaluate the degree of bias and accuracy linked to judgments of the overall quality of the interactions on the SIEM, but multilevel modeling (MLM) for analyses focused on the dyad members’ individual ratings on the FIS due to the hierarchical structure of these data (i.e. interaction participants nested within dyads). In addition, all analyses evaluated whether the diagnostic status of the individuals within the interactions moderated the degree of directional bias and tracking accuracy observed. Analyses were completed using SPSS Version 29 (IBM SPSS Inc., 2022), and all syntax and output for the analyses can be accessed on the Open Science Framework: https://tinyurl.com/4pxz2d7p.

## Results

### Observers’ perceptions of the interaction quality using the SIEM

Table 1 displays the means and 95% Confidence Intervals for observers’ ratings of interaction quality across the three dyad compositions. Dyad composition had a significant effect on observers’ enjoyment of the interaction (*F*(2, 99.81) = 5.92, *p* = 0.004) and the extent to which they perceived the interaction as smooth (*F*(2, 100.43) = 4.03, *p* = 0.02), but not on their perceptions of the interaction’s awkwardness (*F*(2, 100.06) = 1.95, *p* = 0.15) or intimacy (*F*(2, 105.01) = 0.54, *p* = 0.59). Post hoc comparisons revealed that observers rated NA-NA interactions as smoother (*p* = 0.024) and more enjoyable (*p* = 0.004) than A-NA interactions. Observer ratings did not significantly differ between the other levels of dyad composition.

**Table 1.** Means [and 95% confidence intervals] for observers’ ratings of the interactions across the three dyad compositions.

Variable	NA-NA Pairing	A-NA Pairing	A-A Pairing
Observers’ Enjoyment*	4.83 [4.44, 5.22]	3.90 [3.51, 4.29]	4.17 [3.78, 4.55]
Participants’ Enjoyment*	5.38 [5.09, 5.66]	4.85 [4.57, 5.14]	5.01 [4.73, 5.29]
Smoothness*	5.13 [4.85, 5.41]	4.60 [4.32, 4.88]	4.72 [4.45, 4.99]
Awkwardness	3.79 [3.52, 4.06]	4.17 [3.90, 4.45]	4.00 [3.74, 4.26]
Intimacy	3.17 [2.73, 3.60]	2.88 [2.44, 3.31]	3.14 [2.71, 3.58]
Future Interaction	4.90 [4.60, 5.20]	4.62 [4.32, 4.92]	4.74 [4.45, 5.03]
Disclosure*	5.32 [5.08, 5.55]	4.50 [4.26, 4.73]	4.74 [4.51, 4.98]
Influence	5.34 [5.11, 5.57]	5.11 [4.88, 5.34]	5.19 [4.96, 5.41]
Satisfaction	4.99 [4.71, 5.27]	4.52 [4.23, 4.80]	4.63 [4.35, 4.91]
Pleasantness*	5.41 [5.10, 5.72]	4.79 [4.48, 5.10]	4.84 [4.54, 5.14]

NA = Non-autistic. A = Autistic. * = omnibus F-test significant at *p* < 0.05.

### Observers’ perceptions of dyadic experiences using the SIEM

Dyad composition had a significant effect on observers’ perceptions of how much interaction participants enjoyed the interaction (*F*(2, 99.76) = 3.49, *p* = 0.03), disclosed to their partners (*F*(2, 99.00) = 12.66, *p* < 0.001), and experienced pleasantness (*F*(2, 99.56) = 4.99, *p* = 0.01). Dyad composition did not have a significant effect on observers’ perceptions of interaction participants’ levels of influence (*F*(2, 98.85) = 1.01, *p* = 0.37), satisfaction (*F*(2, 99.92) = 2.96, *p* = 0.06), or intentions to interact with their partner again in the future (*F*(2, 99.30) = 0.88, *p* = 0.42). Post hoc comparisons revealed that observers perceived interaction participants in NA-NA interactions to enjoy the interaction more (*p* = 0.034), disclose more (*p* < 0.001), and experience more pleasantness (*p* = 0.02) than in A-NA interactions. In addition, observers perceived interaction participants in NA-NA interactions to disclose more (*p* = 0. 0.002) and experience more pleasantness (*p* = 0.03) than participants in A-A interactions. Observer ratings did not significantly differ between the other levels of dyad composition.

### Observers’ perceptions of individual participants in the interactions using the SIEM

Table 2 shows that observers perceived interaction participants to have worse SIEM outcomes when their partners were autistic versus non-autistic. They reported that interaction participants enjoyed interactions with autistic partners less, disclosed to them less, found the interactions to be less pleasant and satisfying, and would be less likely to interact with them again in the future. They also perceived autistic participants to disclose less toward their partners overall than non-autistic participants. However, Table 2 also shows that the combination of diagnostic status between partners mattered for observers’ perceptions of their disclosure. Breaking this down, whereas observers perceived NA participants to disclose significantly less toward autistic partners (*b* = −0.73, *SE* = 0.18, *p* < 0.001), they perceived autistic participants to marginally (but not significantly) disclose more toward autistic participants (*b* = 0.33, *SE* = 0.18, *p* = 0.06).

**Table 2.** Effects of diagnostic status on observers’ perceptions of interaction participants.

	Intercept		Effect of Participant’s Dx		Effect of Partner’s Dx		Participant’s Dx × Partner’s Dx Interaction	
	b	*SE*	b	*SE*	B	*SE*	b	*SE*
SIEM Ratings								
Enjoyment	5.02	0.09	−0.04	0.11	−0.33**	0.11	0.68†	0.35
Future Interact	4.72	0.09	0.11	0.11	−0.27*	0.11	0.41	0.37
Disclosure	4.76	0.07	−0.37**	0.10	−0.20*	0.10	1.07**	0.29
Influence	5.19	0.07	−0.20^†^	0.10	0.05	0.10	0.31	0.28
Satisfaction	4.66	0.09	−0.02	0.11	−0.34**	0.11	0.59†	0.35
Pleasantness	4.96	0.10	−0.11	0.11	−0.46**	0.11	0.68†	0.38
FIS Ratings								
Awkwardness	2.28	0.03	0.44**	0.04	−0.13**	0.04	−0.08	0.12
Attractiveness	2.20	0.04	−0.41**	0.05	−0.01	0.05	0.10	0.15
Trustworthiness	2.92	0.03	−0.15**	0.04	−0.06	0.04	−0.04	0.13
Dominance	1.90	0.04	0.03	0.05	0.03	0.05	0.03	0.16
Likable	2.87	0.03	−0.29**	0.04	0.01	0.04	0.03	0.11
Intelligence	2.80	0.05	−0.27**	0.06	−0.11*	0.06	0.09	0.18
Live Near	3.10	0.05	−0.22**	0.06	−0.08	0.06	0.15	0.21
Hangout With	2.22	0.04	−0.30**	0.05	0.09^†^	0.05	−0.11	0.15
Comfort	3.11	0.04	−0.24**	0.05	0.04	0.05	0.02	0.18
Conversation	2.41	0.04	−0.25**	0.05	0.13*	0.05	−0.08	0.17

SIEM = Social Interaction Evaluation Measure. FIS = First Impressions Scale. Dx = Diagnosis. Diagnosis was effect coded such that −0.5 = non-autistic and 0.5 = autistic. *SE* = standard error of unstandardized regression coefficient.

† *p* < 0.10. *p < 0.05. **p < 0.01.

### Observers’ perceptions of individual participants in the interactions using the FIS

Table 2 shows that observers perceived autistic participants to be more awkward and less attractive, trustworthy, likable, and intelligent than NA participants. Observers also reported less social interest toward autistic compared to NA participants on all items. Notably, observers perceived participants to be less awkward and smart when they were interacting with autistic compared to NA partners. They also reported wanting to start a conversation more with participants whose partners were autistic compared to NA.

### Truth and bias model analyses comparing observer ratings to participant ratings

#### SIEM results

Our first Truth and Bias model analyses used hierarchical regression analyses to evaluate whether observers’ ratings of the overall quality of the interactions from the SIEM (i.e. enjoyability, smoothness, awkwardness, and intimacy) aligned with the average of the two interaction partners’ self-reports of the same items and whether dyad type (i.e. the combination of interaction participants’ diagnostic statuses; A-A vs NA-NA vs A-NA) moderated directional bias and tracking accuracy. Observers significantly underestimated the interaction participants’ levels of enjoyment (*b* = −0.94, *SE* = 0.29, *p* = 0.003), smoothness (*b* = −0.73, *SE* = 0.30, *p* = 0.02), and awkwardness (*b* = −0.67, *SE* = 0.29, *p* = 0.025), but overestimated their levels of intimacy (*b* = 1.00, *SE* = 0.28, *p* = 0.001). Moreover, although observers displayed tracking accuracy for smoothness (*b* = 1.23, *SE* = 0.34, *p* < 0.001) and awkwardness (*b* = 1.14, *SE* = 0.27, *p* < 0.001), their ratings did not significantly align with the interaction participants’ averaged ratings of enjoyability (*b* = 0.42, *SE* = 0.29, *p* = 0.16) or intimacy (*b* = −0.31, *SE* = 0.35, *p* = 0.382). Furthermore, dyad type did not significantly moderate directional bias (∆*R*^2^s < 0.06, *p*s > 0.20) or tracking accuracy (∆*R*^2^s < 0.11, *p*s > 0.12) for any of the interaction quality outcomes.

#### FIS ratings

Another Truth and Bias model evaluated whether observers’ FIS trait ratings of the interaction participants aligned with how the interaction participants were perceived by their partners, and whether their ratings of the social interest of interaction participants aligned with the interaction participants’ self-reported social interest. Each analysis also tested whether the diagnosis of the interaction participant and/or their partner moderated directional bias and tracking accuracy. Separate MLMs were specified for each trait/social interest, resulting in 10 analyses.

Table 3 shows the Truth and Bias results for the FIS trait ratings. On average, observers rated participant attractiveness, trustworthiness, likeability, and intelligence significantly lower– and awkwardness and dominance significantly higher– than did the participants’ partners in the interaction. Observers tended to underestimate participants’ ratings of their autistic partner’s attractiveness (*b* = −0.46, *SE* = 0.05, *p* < 0.001), trustworthiness (*b* = −0.36, *SE* = 0.02, *p* < 0.001), likeability (*b* = −0.57, *SE* = 0.04, *p* < 0.001), and intelligence: (*b* = −0.56, *SE* = 0.03, *p* < 0.001), significantly more so than participants’ ratings of their NA partners on the same traits (attractiveness, *b* = −0.13, *SE* = 0.05, *p* = 0.014); trustworthiness, *b* = −0.22, *SE* = 0.03, *p* <0.001; likeability, *b* = −0.31, *SE* = 0.04, *p* < 0.001; and intelligence, *b* = −0.31, *SE* = 0.03, *p* < 0.001). Observers also underestimated autistic participants’ ratings of their partners’ trustworthiness and intelligence (trustworthiness: *b* = −0.32, *SE* = 0.03, *p* < 0.001; intelligence: *b* = −0.49, *SE* = 0.03, *p* < 0.001) significantly more than they did NA participants’ ratings of their partners (trustworthiness: *b* = −0.26, *SE* = 0.02, *p* < 0.001; intelligence: *b* = −0.37, *SE* = 0.03, *p* < 0.001).

**Table 3.** Truth and bias model analyses for individual ratings.

Predictors	Individual ratings of traits											
	Awkwardness		Attractiveness		Trustworthiness		Likeability		Dominance		Intelligence	
	*b*	*SE*	*b*	*SE*	*b*	*SE*	*B*	*SE*	*b*	*SE*	*b*	*SE*
Average Bias and Accuracy												
Avg Directional Bias	−0.36**	0.08	−0.29**	0.04	−0.29**	0.02	−0.44**	0.03	0.18**	0.03	−0.43**	0.02
Avg Tracking Accuracy	0.29**	0.10	0.14*	0.05	0.03	0.04	−0.01	0.06	0.07	0.05	0.07**	0.03
Moderators of Directional Bias												
Participant’s Dx	0.07	0.07	−0.16**	0.04	−0.07**	0.02	−0.13**	0.03	0.00	0.03	−0.12**	0.02
Partner’s Dx	−0.06	0.07	−0.02	0.04	−0.03*	0.02	−0.00	0.03	0.01	0.03	−0.06**	0.02
Participant’s Dx × Partner’s Dx	0.00	0.08	0.01	0.04	−0.01	0.02	0.00	0.03	0.00	0.03	0.00	0.02
Moderators of Tracking Accuracy												
Participant’s Dx	−0.10	0.10	−0.03	0.05	0.03	0.04	0.01	0.05	0.00	0.05	0.07**	0.02
Partner’s Dx	−0.05	0.10	−0.01	0.05	−0.02	0.04	0.03	0.05	−0.04	0.05	0.02	0.02
Participant’s Dx × Partner’s Dx	0.11	0.10	0.02	0.05	−0.03	0.04	0.06	0.06	0.06	0.05	0.02	0.03

Avg. = Average. Dx = Diagnosis. Diagnosis was effect-coded, such that −1 = Not autistic and 1 = Autistic. *SE* = standard error of unstandardized regression coefficient.

* *p* < 0.05. **p < 0.01.

Table 3 also shows that observers only displayed tracking accuracy for participants’ ratings of their partner’s awkwardness, attractiveness, and intelligence, with tracking accuracy for intelligence moderated by the participant’s diagnosis. Observers displayed significantly more tracking accuracy when participants were rating the intelligence of autistic partners (*b* = 0.13, *SE* = 0.03, *p* < 0.001) relative to NA partners (*b* = 0.001, *SE* = 0.04, *p* = 0.988).

Table 4 shows Truth and Bias results for FIS social interest items. On average, observers reported less social interest in participants than did partners in the interaction, and this occurred for all items. Importantly, these mean-level difference effects were moderated by participant diagnosis such that each effect was significantly larger for autistic interaction participants. That is, observers’ social interest in participants was even lower than the participants’ partners’ social interest when the participant was autistic (live near: *b* = −0.15, *SE* = 0.03, *p* < 0.001; hang out with: *b* = −0.69, *SE* = 0.05, *p* < 0.001; start a conversation with: *b* = −0.82, *SE* = 0.04, *p* < 0.001; and their comfort in sitting next to: *b* = −0.34, *SE* = 0.03, *p* < 0.001) versus non-autistic (live near: *b* = 0.04, *SE* = 0.03, *p* = 0.13; hangout with: *b* = −0.40, *SE* = 0.06, *p* < 0.001; start a conversation with: *b* = −0.60, *SE* = 0.05, *p* < 0.001; and their comfort in sitting next to: *b* = −0.12, *SE* = 0.03, *p* < 0.001. Observers also reported significantly less desire to live near interaction participants than did their autistic partners (*b* = −0.10, *SE* = 0.03, *p* = 0.001) versus non-autistic partners (*b* = −0.01, *SE* = 0.03, *p* = 0.749). None of the observers’ social interest ratings displayed rank-order consistency with the partners’ ratings and the diagnoses of the interaction participants and their partners did not moderate any of these effects.

**Table 4.** Analyses for behavioral intention items using the truth and bias model framework.

Predictors	Behavioral intention ratings							
	Living near partner		Hangout with partner		Uncomfortable		Conversation	
	*b*	*SE*	*b*	*SE*	*B*	*SE*	*b*	*SE*
Average Mean-Level Difference and Rank-Order Consistency								
Avg Mean-Level Difference	−0.05*	0.02	−0.55**	0.04	−0.23**	0.02	−0.71**	0.03
Avg Rank-Order Consistency	−0.02	0.02	−0.01	0.05	−0.03	0.03	0.06	0.06
Moderators of Mean-Level Difference								
Participant’s Dx	−0.10**	0.02	−0.14**	0.04	−0.11**	0.02	−0.11**	0.03
Partner’s Dx	−0.04*	0.02	0.04	0.04	0.01	0.02	0.04	0.03
Participant’s Dx*Partner’s Dx	0.03	0.02	−0.05	0.04	0.003	0.02	−0.03	0.03
Moderators of Rank-Order Consistency								
Participant’s Dx	−0.02	0.02	−0.09	0.05	−0.05	0.03	−0.10	0.06
Partner’s Dx	−0.03	0.02	0.06	0.05	−0.02	0.03	0.004	0.06
Participant’s Dx × Partner’s Dx	−0.01	0.02	−0.05	0.05	0.03	0.03	−0.04	0.06

Avg. = Average. Dx = Diagnosis. Diagnosis was effect-coded, such that −1 = Not autistic and 1 = Autistic. *SE* = standard error of unstandardized regression coefficient.

* *p* < 0.05. **p < 0.01.

## Discussion

Autistic people have described themselves as experiencing an increased intra-community connectedness and a more natural rapport with other autistic people (Botha et al., 2020) compared to with non-autistic (NA) people. In line with the double-empathy problem (Milton, 2012), recent studies have provided empirical corroboration of these anecdotal accounts (e.g. Chen et al., 2021; Crompton, Ropar, wt al., 2020; Heasman & Gillespie, 2019; Morrison et al., 2020), collectively indicating better connection and communicative understanding during interactions with autistic relative to NA social partners. However, because markers of social connection may differ for autistic people (Rifai et al., 2022), it remains unclear whether NA observers detect greater connection and affiliation between autistic partners relative to cross-neurotype interactions. This study investigated whether NA adults detect the reduced social quality reported by autistic and NA participants in previously recorded (Morrison et al., 2020) mixed interactions (i.e. one autistic and one NA adult) relative to same-neurotype interactions (i.e. two autistic people or two NA people). Such a finding would indicate that the double-empathy problem is noticeable to NA observers, potentially providing an avenue for improving communication and understanding between autistic and NA people. Recognition of the double empathy problem, and more specifically that mixed interactions (rather than autistic interactions) are perceived as the least successful by NA observers, could provide a foundation for educating NA people about social communication differences in autism and ways of conceptualizing autistic sociality that does not rely on deficit framing.

Overall, results demonstrated that some aspects of the double-empathy problem are perceived by external NA observers. For example, NA observers rated mixed interactions of autistic and NA partners lower than NA-NA ones on two of the four items assessing social interaction quality and three of six assessing their perceptions of partners’ feelings and behaviors during the interaction. In contrast, they did not rate conversations between two autistic partners lower than those between two non-autistic ones on any of the four metrics of interaction quality, or on perceptions of the participants’ interest in future interaction with their partner or their influence on the interaction. These findings suggest that NA observers detected elements of the double empathy problem (Milton, 2012), as they tended to perceive greater disjuncture between mixed dyads of autistic and NA people than between interactions where partners were both NA or both autistic.

Furthermore, observers perceived that only NA participants disclosed more to NA partners, with a marginal effect in the opposite direction of autistic participants disclosing more to autistic partners. This pattern corresponds with what the NA and autistic participants in the interactions themselves reported (Morrison et al., 2020), and with previous research showing that autistic people often demonstrate increased rapport with each other relative to with NA people (Crompton, Sharp, et al., 2020). Taken together, these findings not only suggest that autistic adults may feel an increased ability to share authentically when interacting with other autistic people, but that this difference is noticeable to those outside of the interaction. Autistic people may feel less pressure to mask their autistic characteristics when in the presence of other autistic people, potentially leading to increased openness and rapport. This interpretation may also relate to why sample values of social interaction quality were lowest for mixed dyads rather than those between two autistic people. First impression ratings were still lowest for autistic participants, though, regardless of the dyad composition. Thus, any increased interpersonal connectivity experienced by two autistic participants did not translate into more positive evaluations of the autistic people within them, suggesting an undervaluing of autistic sociality by NA observers.

Individually, participants were rated less favorably on five items of the SIEM when interacting with an autistic person relative to an NA person. This suggests that evaluations of people are shaped in part by who they are interacting with; in this case, perceptions of autistic peoples’ partners, whether NA or autistic, may have been negatively affected by perceptions of autistic sociality within the interaction. This may be a form of stigma by association where the decreased perceived social status of the autistic participant affects the perception of their interaction partner. In mixed interactions, observers may also be detecting aspects of disjunction between NA and autistic partners and attributing some of the reduction in social quality to the NA partner. Observers in this study were NA and may have privileged or imposed neurotypical, normative markers of social connection (e.g. fluidity, eye contact, reciprocity, displays of positive affect) that are not as apparent in mixed interactions and conclude that NA participants are less socially successful than those in same-neurotype interactions in which those normative markers are more likely to occur.

Relatedly, observers perceived autistic and NA participants’ intelligence to be higher when they were interacting with NA partners and lower when interacting with autistic ones. Higher ratings of autistic intelligence when interacting with a NA partner may indicate that they are adapting their social behavior to meet NA social preferences and expectations, resulting in more positive assessments from NA observers. This real-time adaptation to NA social norms is often made to be the responsibility of autistic people, including in social skill training programs (Bottema-Beutel et al., 2018). Such an interpretation is also consistent with autistic masking as adaptation to non-autistic social standards in order to avoid marginalization, stigmatization or discrimination (Botha et al., 2020; Pearson & Rose, 2021). Furthermore, lower intelligence ratings of NA participants when interacting with autistic partners suggests that NA observers perceive them less favorably when interactions do not meet normative standards of social success.

Interestingly, participants were also rated as less awkward when interacting with autistic relative to NA partners, and observers reported more interest in starting a conversation with participants when their partner was autistic. The first of these effects may be the result of social comparison on the part of NA observers. The high ratings of awkwardness of autistic partners may have reduced their perception of awkwardness of their partners. For the second, observers may have detected greater conversational effort of interactional participants when interacting with autistic partners and judged them to be better conversation partners as a result.

NA observers provided less favorable impressions of participants and lower social interest in them than did the actual participants within the interactions, and this effect was larger for autistic participants. This may suggest that NA people may form more positive impressions of autistic people when directly interacting with them than through passive observation. However, this study was not designed to test this, and future work is encouraged to explore whether direct contact between NA and autistic people can reduce biases over time and mitigate the double empathy problem. If so, such evidence would support efforts to use positive interpersonal/intergroup contact to address negative attitudes toward autistic people. A meta-analysis on using intergroup contact to address the stigmatization of mental illness (Maunder & White, 2019) found that using face-to-face, imagined, video, or presentation-based contact reduced stigma relatively equally, but the effects of vicarious contact– the act of watching members of outgroups interact with other ingroup or outgroup members– had limited evidence to assess benefits. This study suggests that for non-autistic people, vicarious contact does not substitute for direct contact in terms of forming positive attitudes toward autistic people. More importantly, biases which are being formed or sustained through vicarious interaction (observing autistic people interacting with non-autistic people) may impede NA people’s willingness to engage with autistic people before they directly engage with autistic people themselves. However, educational programming featuring autistic testimony has shown promise for reducing explicit (but not implicit) biases about autism (Jones, DeBrabander, et al., 2021) and for increasing social interest between autistic and non-autistic social partners during real interaction (Jones, Morrison, et al., 2021). Future work is encouraged to examine whether sustained interaction between autistic and NA people over time may reduce aspects of the double empathy problem, facilitate social connection and understanding, and lower stigma.

Several ratings provided by NA observers aligned more with those provided by NA participants in the interactions than those provided by autistic participants. Not only did their ratings of participant trustworthiness and intelligence correspond more with NA participant ratings than autistic participant ratings, but their social interest ratings in participants lagged behind those provided by participants in the interaction, including autistic ones. Specifically, they expressed less social interest on all items than did autistic and NA participants in the interactions. Such a finding is counter to theories of reduced social interest or motivation in autism (Chevallier et al., 2012) and suggests that autistic social interest may meet or in some scenarios exceed the social interest of NA people. For instance, prior work has shown that autistic adults express greater social interest than NA adults in other autistic people (DeBrabander et al., 2019; Morrison et al., 2020). Future research should examine whether NA people underestimate or misperceive signs of autistic social interest, which could occur if autistic social interest is expressed differently or is less observable to NA perceivers (Edey et al., 2016; Sheppard et al., 2016). Given the nature of the double empathy problem (Milton, 2012), future studies should also include autistic observers to determine whether findings may differ between autistic and NA observers. For instance, autistic observers may detect greater social connection between autistic partners than the NA observers did in this study and may provide ratings that better align with those provided by autistic relative to NA participants in the interactions. Research that accounts for the effects of masking is also needed.

This study should be interpreted in the context of several limitations. First, the sample of interaction participants was limited to the 86 of the 125 participants from the original study (Morrison et al., 2020), as we only included videos from those consented for them to be viewed by other people. A larger sample of interaction and observer participants may have been better powered to detect more sensitive effects, and thus non-significant differences between ratings of dyad types should not be interpreted as indicating equivalence. In addition, the sample of interaction and observer participants also lacked representativeness. To isolate effects of diagnosis and avoid confounding effects of gender that would have required a prohibitively large sample to examine (see Morrison et al., 2020), the interaction sample consisted entirely of self-identifying males. Participants were also largely White and highly educated, so it is unclear how interaction dynamics may have differed with a more diverse sample. Observers predominantly self-identified as female and, although observer gender has largely not produced effects in prior related studies (Morrison et al., 2019; Sasson & Morrison, 2019), results here may have differed with a more balanced gender composition. Future research needs to account for intersectional effects, including race and gender, on interactions between autistic and NA people and on observer perceptions. Furthermore, because the sample of observers consisted of psychology students from a campus with a large autistic population (Hoffman, 2016), they may have greater familiarity with autism than the general population, and some may have even encountered the concept of “double empathy” or research conducted by this laboratory. Such familiarity, however, would reduce biases toward autistic differences or increase social desirability effects, and thus the findings reported here are likely a conservative estimate relative to other NA observer samples.

Finally, because there is debate about using parametric statistics with individual Likert-type items like those on the SIEM and FIS (Stevens, 1946; Velleman & Wilkinson, 1993), future work would benefit from using multi-items measures of each construct with more response options. These measures also require force-choiced responses, which reduces missing data but can increase measurement error. In addition, it is possible that autistic and non-autistic participants interpreted items on these measures differently, as has been found for self-report of autistic traits (Gernsbacher et al., 2017). Future studies should more explicitly examine whether autism knowledge and familiarity in NA observers affects the patterns reported here.

In sum, this study reveals that NA observers both detect and demonstrate aspects of the double empathy problem when evaluating interactions among and between autistic and NA adults. They detected that mixed interactions between autistic and NA people were lower in social quality across several dimensions than interactions between two NA people but did not report any significant differences in social quality between NA dyads and autistic dyads. They also perceived that only NA participants disclosed more to NA partners, with a marginal effect of autistic people disclosing more to autistic relative to NA partners. However, several of their observations aligned more closely with ratings provided by NA compared to autistic participants within the interactions, they underestimated trust and intelligence ratings made by autistic participants, and they reported lower social interest in participants than did the autistic and non-autistic people in the interactions. Finally, NA ratings provided by individuals within interactions with autistic people were generally more favorable than those provided by NA observers, suggesting that direct personal contact may reduce stigma and improve NA impressions of autistic people.

## Notes

1. There is very little quality-of-life literature currently that has been autistic-led, and based on what autistic people value for their own lives and this is a severe limitation of quality-of-life literature. There is an emerging focus on autistic-led quality of life from projects which are forthcoming (Milbourn et al., 2022).
